# Extracts of *Rheum palmatum* and *Aloe vera* Show Beneficial Properties for the Synergistic Improvement of Oral Wound Healing

**DOI:** 10.3390/pharmaceutics14102060

**Published:** 2022-09-27

**Authors:** Lena Katharina Müller-Heupt, Nadine Wiesmann, Sofia Schröder, Yüksel Korkmaz, Nina Vierengel, Jonathan Groß, Rolf Dahm, James Deschner, Till Opatz, Juergen Brieger, Bilal Al-Nawas, Peer W. Kämmerer

**Affiliations:** 1 Department of Oral- and Maxillofacial Surgery, University Medical Center Mainz, Augustusplatz 2, 55131 Mainz, Germany; 2 Department of Otorhinolaryngology, University Medical Center Mainz, Langenbeckstr. 1, 55131 Mainz, Germany; 3 Department of Periodontology and Operative Dentistry, University Medical Center Mainz, Augustusplatz 2, 55131 Mainz, Germany; 4 Department of Chemistry, Johannes Gutenberg-University, Duesbergweg 10-14, 55128 Mainz, Germany; 5 Beratung für Informationssysteme und Systemintegration, Gärtnergasse 1, 55116 Mainz, Germany

**Keywords:** *Aloe vera*, broth microdilution, fibroblasts, phenomenological combination index, *Porphyromonas gingivalis*, promigratoy effect, *Rheum palmatum* root extract, scratch assay, synergy, wound healing

## Abstract

Various local and systemic factors compromise oral wound healing and may lead to wound dehiscence, inflammation, or ulcers. Currently, there is a lack of topical therapeutical options. Thus, this study aimed to investigate the effect of *Aloe vera* (AV) and *Rheum palmatum* root (RPR) on oral wound healing capacity in vitro. The effect of AV and RPR on human primary fibroblast viability and migration was studied by measuring metabolic activity and gap closure in a scratch assay. Furthermore, cell cycle distribution and cytoskeletal features were analyzed. Antimicrobial activity against the oral pathogen *Porphyromonas gingivalis* was evaluated by broth microdilution assay. AV and RPR increased fibroblast migration after single agent treatment. Synergistic effects of the plant extract combination were observed regarding cellular migration which were confirmed by calculation of the phenomenological combination index (pCI), whereas the cell cycle distribution was not influenced. Furthermore, the combination of AV and RPR showed synergistic antibacterial effects as determined by the fractional inhibitory concentration index. This study demonstrated that the combination of AV and RPR can promote the migration of human primary fibroblasts in vitro and exert antimicrobial efficacy against *P. gingivalis*, suggesting these compounds for the topical treatment of wound healing disorders.

## 1. Introduction

Wound healing in the oral cavity consists of a sequence of complex biological processes in direct proximity to oral fluids containing millions of microorganisms [1,2]. More than 700 bacterial species were identified in the oral cavity, some of them known to be linked with oral diseases such as caries or periodontitis [3,4]. Wound healing is a complex process orchestrated by different cell populations. Irrespective of the type of wounded tissue, tissue repair follows four phases: coagulation and haemostasis, inflammation, proliferation, and remodelling [5]. Fibroblasts are one of the decisive cell types for oral wound healing. In the scope of tissue repair, resident fibroblasts begin to proliferate and migrate into the fibrin clot that was formed. By deposition of extracellular matrix, contraction of the wound, and breakdown of the fibrin clot fibroblasts are capable of shifting the wound microenvironment from the inflammatory to the growth state [6]. Thus, fibroblast viability and migratory potential are important aspects, in addition to prevention of excessive inflammation, that play a role in oral wound healing. Furthermore, a balanced inflammatory response seems to play a pivotal role in providing optimal wound healing of the oral mucosa [7,8].

Healthy oral mucosa has the ability to heal with minimal to no scars [9], but wound healing in the oral cavity can be disturbed by a variety of factors, such as smoking, local toxins, head or neck irradiation, xerostomia, infection, or hypoperfusion [1,10,11,12,13]. Furthermore, general factors such as antiresorptive medication, alcoholism, diabetes, age, nutritional deficiency, chemotherapy, HIV, or corticosteroids negatively affect physiologic oral wound healing [14,15,16]. Clinical manifestations can be wound dehiscence, granuloma formation, ulcers, pus formation, or formation of granulation tissue [1]. In a clinical context, wound healing and inflammatory disbalances negatively impact patient health and quality of life. Oral lichen planus (OLP) and oral mucositis are examples for such clinical disease patterns. OLP, a chronic inflammatory disease of the oral mucosa, affects around 2% of the adult population and increases the risk for malignant transformations and the development of oral squamous cell carcinoma [17,18,19,20]. Patients with OLP often suffer from burning sensations or pain, especially if erosive mucosal lesions occur.

A topical preparation containing dexpanthenol and silbiol showed no beneficial effect on mucosal wound healing in a rat model [21]. Furthermore, neither dexpanthenol nor *Aloe vera* alone displayed prophylactic potential for patients undergoing mucosal irradiation [22]. Currently, there is a lack of topical preparations sufficiently improving oral wound healing [23].

*Porphyromonas gingivalis* is a gram-negative anaerobic bacterium and a common pathogen of the oral microbiota. It is capable of colonizing the oral epithelium [24] and closely associated with periodontitis, but can exist within the host epithelium even in patients without periodontitis [24]. *P. gingivalis* is known to compromise cell migration due to downregulation of integrin beta-3 and -6 [25] and their capsular polysaccharides and virulence factors such as gingipains were found to hinder wound healing [26,27].

Based on the above-mentioned clinical indications, the aim of this study was to evaluate the potential two plant extracts, *Aloe vera* (AV) and *Rheum palmatum* root (RPR) extract to (a) improve oral tissue regeneration by promoting fibroblast migration and (b) to exert antibacterial activity against *P. gingivalis* to maintain the inflammatory balance of oral tissues.

AV is traditionally used for its versatile biological activities such as its capacity to accelerate wound healing, its anti-inflammatory activity, and its immunomodulatory effects [28,29,30]. Furthermore, AV showed promising results in clinical studies in patients with OLP, oral submucous fibrosis, burning mouth syndrome, candida associated denture stomatitis, xerostomic patients, and recurrent aphtous stomatitis [31].

RPR extract, known as Da Huang in traditional Chinese medicine, is a medicinal plant from Asia and the Middle East. RPR is known to exert anti-inflammatory and anti-fibrotic effects [32,33] and was shown to be an effective antimicrobial agent against oral pathogens such as *P. gingivalis* [34,35,36]. The major antimicrobial activity is suspected to partially originate from the presence of anthraquinones, especially from the anthraquinone rhein [34,35]. Furthermore, Liao et al. suggested potential oral wound healing properties since epithelial cell proliferation was increased by RPR [35].

Thus, our study aimed to evaluate the potential of AV and RPR and a combination thereof (AVRPR) to improve oral tissue regeneration and to exert antibacterial properties. We found increased fibroblast migration and antibacterial properties suggesting these compounds for the topical use as mouthwash or mouthrinse for patients with a compromised wound healing capacity.

## 2. Materials and Methods

### 2.1. Cell Isolation and Cell Culture

Primary human fibroblasts (hereinafter referred to as “fibroblast”) were isolated from mucosa obtained from patients who underwent surgery at the Department of Otorhinolaryngology, University Medical Center Mainz, Mainz, Germany. This study was performed in agreement with the declaration of Helsinki on the use of human material for research. In accordance with the ethics committee of Rhineland-Palatinate, patients agreed with the scientific use of the surplus material and no further approval of the medical ethics committee was required as the fibroblasts were used anonymously. Tissue samples were cut into small pieces of approximately 2 × 2 mm with a sterile disposable scalpel. Prior to cell isolation, the tissue pieces were stepwise disinfected in 70% ethanol, in Sterilium^®^ classic pure (Bode Chemie GmbH, Hamburg, Germany), and again in 70% ethanol. Then they were transferred to 5–10 mL (depending on the amount of tissue) 0.5% protease solution (P6141, Sigma-Aldrich, St. Louis, MO, USA) in phosphate buffered saline (PBS; Sigma-Aldrich, St. Louis, MO, USA) and incubated overnight at 4 °C. The next day, the protease solution was incubated with shaking for further 15 min at 37 °C. The sample was then passed through a cell sieve (EASYstrainer^TM^ 70 µm sterile, Greiner bio-one, Kremsmünster, Austria) with the help of a cell scraper (Falcon^®^, Corning, NY, USA). Cells were pelleted by centrifugation at 1500 rpm = 470 rcf (470× *g*) for 5 min, transferred to cell culture medium, and seeded into small cell culture flasks with 25 cm^2^ grow area. Cells were characterized morphologically and were used at most until passage 10 to ensure primary identity. Cells were maintained in DMEM/Ham’s F12 (Gibco, ThermoFisher Scientific, Waltham, MA, USA) supplemented with 10% fetal calf serum and antibiotics (10,000 U/mL penicillin and 10 mg/mL streptomycin, Sigma-Aldrich, St. Louis, MO, USA) at 37 °C in 5% CO_2_.

### 2.2. Microorganisms and Culture Conditions

*P. gingivalis* (DSM No. 20709, ATCC 33277) was obtained from the German Collection of Microorganisms and Cell Cultures (DSMZ) and cultivated on Schaedler agar (Becton-Dickinson, Heidelberg, Germany) to obtain 48-h-old cultures for further processing. A working aliquot was held at −70 °C and used for weekly sub-culturing. Anaerobic cultivation was performed under anaerobic conditions (90% N_2_, 10% CO_2_, 10% H_2_) in an anaerobic jar system (Anoxomat Mart II, Mart Microbiology BV, Lichtenvoorde, The Netherlands).

### 2.3. Preparation of RPR/AV Solutions for Cell Culture Experiments

RPR and AV were obtained from Paninkret (Paninkret, Pinneberg, Germany). The substances were dissolved in sterile filtered, deionised, and autoclaved water and the solutions were adjusted to concentrations of 10–2048 mg/L. Solutions were freshly prepared for each experiment. The identity of the RPR extract used in this study was identified according to the European Pharmacopeia and the European Union herbal monograph on *Rheum palmatum* L. and *Rheum officinale* Baillon, radix (EMEA/HMPC/189624/2007). AV was obtained from *Aloe barbadensis* Miller and its identity was identified according to the European Union herbal monograph on *Aloe barbadensis* Mill. and on *Aloe* (various species, mainly *Aloe ferox* Mill. and its hybrids), folii succus siccatus.

### 2.4. HPLC (High-Performance Liquid Chromatography) Analysis

Analysis of AV was performed on an Agilent Infinity II 1260 system with a diode array detector. An ACE C18-PFP column (150 mm × 4.6 mm, 3 μm, 40 °C) was applied as stationary phase. A gradient mixture of acetonitrile and water (containing 0.1% formic acid) with a constant flow rate of 1.0 mL/min was used as an eluent with the following linear gradient elution program: 0 min: 99% H_2_O and 1% MeCN, 30 min: 5% H_2_O and 95% MeCN, 40 min: 5% H_2_O and 95% MeCN. For HPLC analysis, the material was dissolved in a 1:1 (*v*/*v*) MeCN/H_2_O solution resulting in a concentration of 1.02 mg/mL. The resulting sample was filtered over a Macherey-Nagel syringe filter with a PTFE membrane (0.2 µm pore size) prior to injection with an injection volume of 3 μL.

The HPLC analysis of RPR (Paninkret, Pinneberg, Germany) containing the UV chromatogram at 254 nm and the corresponding peak list was published in a previous study [34].

### 2.5. NMR (Nuclear Magnetic Resonance) Analysis

NMR spectra were recorded on a Bruker (Billerica, MA, USA) Avance-III (^1^H-NMR: 600 MHz, ^13^C-NMR: 151 MHz) spectrometer. Chemical shifts were referenced to the residual solvent signal (D_2_O: 4.79 ppm for ^1^H-NMR) and reported in parts per million (ppm) relative to tetramethylsilane (TMS).

### 2.6. Cellular Viability

Fibroblasts were seeded into a 96-well-plate (10,000 cells/well in 250 μL cell culture medium) and were given time to adhere overnight. After 24 h, cells were treated with 2 mg/L to 200 mg/L RPR (Paninkret Chem.-Pharm. Vertriebsgesellschaft mbH, Pinneberg, Germany) and/or 10 µg/mL to 2000 µg/mL AV extract (Paninkret Chem.-Pharm. Vertriebsgesellschaft mbH). The concentrations of RPR to be tested regarding their effect on cellular viability were chosen according to previous experiments with *R. palmatum* root extract and a reported minimum inhibitory concentration of RPR against *P. gingivalis* in the range of 4 mg/L [34]. Thus, cellular viability assays with different human primary fibroblasts above and below this concentration were performed to evaluate the cytotoxicity of RPR. Regarding AV, MIC values of 50 µg/mL on *P. gingivalis* strain ATCC 33,277 were reported [37], which could not be replicated in our study. Nevertheless, we chose concentrations below and above for the cellular viability assays with different human primary fibroblasts.

The substances were suspended in cell culture medium without FCS. Untreated cells that were equally maintained in medium without FCS served as control. After 24 h treatment, the medium was exchanged for medium with 10% AlamarBlue™ cell viability reagent (ThermoFisher Scientific, Waltham, MA, USA) and the cells were incubated for 4 h at 37 °C. The AlamarBlue assay is based on the change of the blue color of the non-fluorescent indicator dye (resazurin) after acceptance of electrons, which, passing from the oxidized state to the reduced state, becomes a fluorescent pink compound [38]. Fluorescence was measured on a fluorescence microplate reader (Fluoroskan Ascent Microplate reader, ThermoFisher Scientific, Waltham, MA, USA). Results were given as relative fluorescence using a 538 nm excitation filter and a 600 nm emission filter, normalized to untreated control.

### 2.7. Migration Assay

To analyse the cellular migratory capacity, culture-inserts in a 35 mm μ-dish-system (ibidi^®^ GmbH, Munich, Germany) were used. Twenty-four hours before treatment, 28,000 cells were seeded in each chamber within 70 µL of cell culture medium and incubated overnight. After removal of the insert the cells were treated with the indicated amount of RPR and/or AV. The substances were suspended in cell culture medium without FCS. Untreated cells that were equally maintained in medium without FCS served as control. The defined scratch in the cell layer was photographed after every 24 h for 10 d (24–240 h). For computer-assisted objective evaluation of the gap closure the “T-scratch”-software (https://www.cse-lab.ethz.ch/software/, accessed on 21 September 2020) was used.

### 2.8. Calculation of the Phenomenological Combination Index (pCI)

To establish and determine notions of synergy and antagonism regarding cell migration [39], a method based on the median effect principles was established and derived from generalized mass action considerations by fundamental considerations. Due to our setup, however, by working with plant extracts with different active components and well-defined cell culture samples, the strict principle-based index of Chou et al. [39] was modified into a phenomenological index pCI in order to reflect these differences.

As such, we used the quotients pCI_AV_ = f_AVRPR_/f_AV_ and pCI_RPR_ = f_AVRPR_/f_RPR_ which describe the individual relations of the two extracts AV and RPR. According to the experimental setup, quotients <1 are sufficient to assume synergies and show the effects of faster closure in comparison of the extracts and thus a better phenomenological efficiency under the above assumptions. To discuss more sophisticated phenomenological indices, experimental data and statistics must be enhanced. Please see Supplementary Materials for a more detailed mathematical derivation and discussion.

### 2.9. Cell Cycle Analysis

For cell cycle analysis, cells were treated as in the protocol described above for 48 h. AV and RPR were suspended in cell culture medium without FCS. Untreated cells that were equally maintained in medium without FCS served as control. For isolation, cells were washed with PBS and finally suspended in 500 μL PBS and 4.5 mL 70% ice-cold ethanol. After incubation for at least 2 h at −20 °C, cells were washed with washing buffer [0.2% (*v*/*v*) Triton X-100, 1% BSA in PBS] and transferred to propidium iodide staining solution [0.1% (*w*/*v*) RNAse A, 5 μg/mL PI in PBS]. The staining was evaluated with the FACSCanto™ flow cytometer (Becton Dickinson, Franklin Lakes, NJ, USA) and analyzed with the associated BD FACSDiva™ software.

### 2.10. Actin Staining

For actin staining, 10,000 fibroblasts were seeded into 4-well-chamber-slides (BD Falcon™ 4 chamber polystyrene vessel tissue culture treated glass slide, Becton, Dickinson and Company, Franklin Lakes, NJ, USA). After 24 h, the cells were treated with RPR, AV, or the combination for 24 h. The substances were suspended in cell culture medium without FCS. Untreated cells that were equally maintained in medium without FCS served as control. After treatment, cells were fixed with 4% paraformaldehyde (Sigma-Aldrich, St. Louis, MO, USA) for 10 min. Then, slides were washed three times with PBS. Cells were incubated in a mixture of 0.1% triton x (Sigma-Aldrich, St. Louis, MO, USA) for 5 min and washed again three times with PBS. Then 1% PBS/BSA (bovine serum albumin) (Sigma-Aldrich, St. Louis, MO, USA) was put on the slides for 30 min. Twenty µL of phalloidin, Thermo Fisher Scientific Inc., Waltham, MA, USA) was dissolved in methanol and mixed with 5.98 mL PBS for fibroblast staining. After 60 min, washing with PBS was repeated three times. For nucleus staining, bisbenzimide (Sigma-Aldrich, St. Louis, MO, USA) was pipetted on the slides for one minute. After twice more washing with PBS, fluorescence mounting medium (Dako North America Inc., Carpinteria, CA, USA) was put on the slides and they were covered with a cover glass. Pictures were taken after drying with the Keyence BZ-9000 microscope and matching BIOREVO BZ-9000 software (Keyence Corporation, Ōsaka, prefecture Ōsaka, Japan). Cell length and fluorescence were evaluated using ImageJ software [40]. Cell length was measured by the measuring tool using the scale as a reference. To assess the intensity of the phalloidin staining, fluorescent pictures were converted to 16-bit pictures and the mean grey value was measured and divided by the cell count.

### 2.11. Broth Microdilution Assay

MIC was determined by serial microdilution as described before [34] and according to the Clinical and Laboratory Standards Institute (CLSI) guidelines [41]. AV was prepared as described in 2.3. whereas RPR was dissolved in 100 mL 50% aqueous ethanol (Carl Roth), since the stock concentration of 2048 mg/L was not fully soluble in water.

Bacterial suspensions of 1 × 10^7^ colony-forming units (0.5 McFarland standard) were used. Of these suspensions, 1 mL was added to 9 mL Wilkins Chalgrens broth (Merlin GmbH, Bornheim-Hersel, Germany). Microdilution was performed using sterile 96-well plates (Greiner Bio-One GmbH, Frickenhausen, Germany) with a final test volume of 100 μL per well. Column one served as negative control (sterile broth only), whereas column two served as positive control (bacterial suspension only). A serial dilution of each test substance was performed, starting in column four as quadruples. A solution with either 2048 mg/L AV or 2048 mg/L RPR or 1024 mg/L RPR and 1024 mg/L AV was used as start concentration in column four. After the potentially antimicrobial agent had been diluted, the same volume of the standardized bacterial suspension was added to each well (50 µL). A solvent control (50% distilled water/50% ethanol) was performed in the same way on a separate plate to exclude antibacterial activity of the solvents. After an incubation period of 48 h for *P. gingivalis* at 37 °C, the plates were inspected. The MIC was determined as the lowest concentration where no visible growth was seen in the wells. Each test was repeated three times.

### 2.12. Calculation of the Fractional Inhibitory Concentration Index (FICI)

To calculate antibacterial synergistic effects of AVRPR, fractional inhibitory concentration index (FICI) was calculated using the following formula:

FICI = (MIC_A_^combi^/MIC_A_^alone^) + (MIC_B_^combi^/MIC_B_^alone^)

According to Odds et al. “Synergy” is defined as a ≥4-fold reduction in the MICs of both compounds in combination compared to their MICs alone (FICI ≤ 0.5); “no interaction” when the MICsyn of one of the compounds remained in the range of 1/2× to 4× MIC (FICI > 0.5–4); and “antagonism” when the MICsyn of both compounds is, at least, 4-fold higher than compared to the activity of the compounds alone (FICI > 4.0) [42].

### 2.13. Statistical Analysis

Unless stated otherwise, results were expressed as mean +/− standard deviation (SD). Kolmogorov–Smirnov normality test was used to determine if data sets were well-modelled by a normal distribution. In case of normal distribution, the ANOVA test was used, elsewhere the Kruskal–Wallis test was applied. A *p*-value of less than 0.05 was considered as statistically significant. Correction for multiple comparisons was done by Bonferroni. All statistical analyses were performed using Prism 6.0 for Windows (GraphPad Software Inc., La Jolla, CA, USA). Statistical significance denoted as * *p* < 0.05, ** *p* < 0.01, *** *p* < 0.001.

## 3. Results

### 3.1. Chemical Analysis of AV and RPR

The HPLC analysis showed that the AV sample consisted almost exclusively of water-soluble aliphatic compounds (Figure S1). The NMR analysis revealed the main components to be acemannan, glucose, malic acid, and citric acid (Figures S2 and S3). A quantitative analysis thereof was provided be the vendor (attached as Supplementary Materials, Table S1).

The presence of anthraquinones and a rhein concentration of 5% in RPR was indicated by HPLC as presented before [34]. The UV chromatogram and the peak list of RPR and the extracted ion current chromatograms (EIC) of the five most abundant anthraquinones and their glycosides in negative ionization mode (ESI–) are depicted in a previous study [36].

### 3.2. Cellular Viability

**Figure 1:**
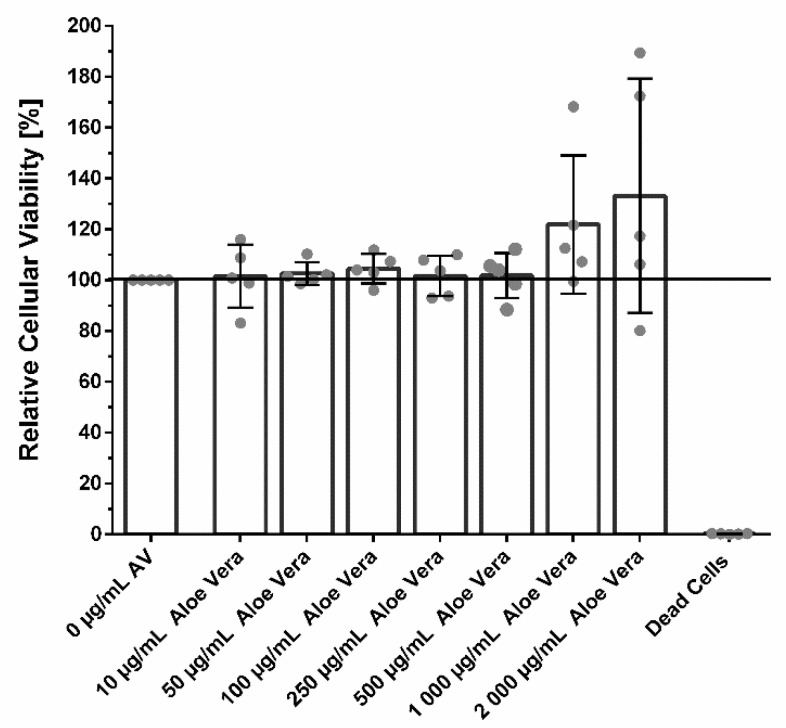
Relative cellular viability of fibroblasts treated with different concentrations of AV ranging from 10 µg/mL to 2000 µg/mL for 24 h. Cellular viability was slightly increased by AV concentrations above 1000 µg/mL. Different fibroblast cultures reacted differently towards AV concentrations above 1000 µg/mL as indicated by high standard deviation. Shown are means ± SD and all individual values are shown in form of a scatter blot, N = 5 with fibroblasts from different patients. One-way ANOVA, comparison of each treatment group with untreated control group, no significant differences were found.

It was shown that AV was non-toxic in all concentrations ranging from 10 µg/mL to 500 µg/mL. Higher concentrations of 1000 µg/mL AV slightly increased the cellular viability without reaching significance (Figure 1). Concentrations of 2000 mg/mL slightly reduced cellular viability of fibroblasts from some patients, while in others cellular viability was increased compared to untreated control cells. The scatter blot in Figure 1 shows that fibroblast cultures from different patients reacted differently to the treatment with AV.

**Figure 2:**
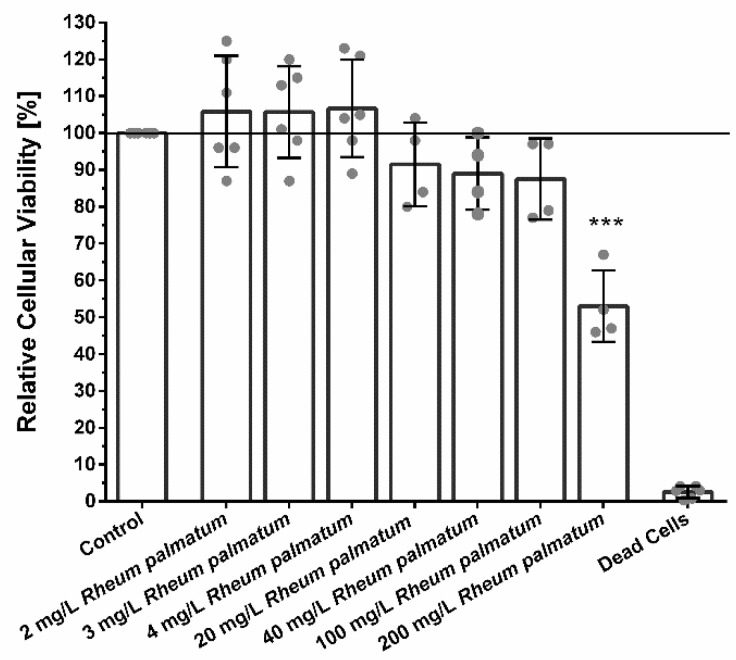
Relative cellular viability of fibroblasts treated with different concentrations of RPR ranging from 2 mg/L to 200 mg/L. Cellular viability of fibroblasts was not significantly influenced by RPR concentrations ranging from 2–100 mg/L. Concentrations ranging from 20–100 µg/mL RPR slightly reduced cellular viability. Higher concentrations of 200 µg/mL RPR significantly decreased cellular viability. Shown are means ± SD and all individual values are shown in form of a scatter blot, N ≥ 4 with fibroblasts from different patients. One-way ANOVA, comparison of each treatment group with untreated control group, *** *p* < 0.001.

Low concentration of <100 µg/mL RPR did not show significant cytotoxic effects on fibroblasts. Higher concentrations of 200 µg/mL RPR significantly decreased the cellular viability and metabolism as shown in Figure 2.

### 3.3. Cell Migration

**Figure 3:**
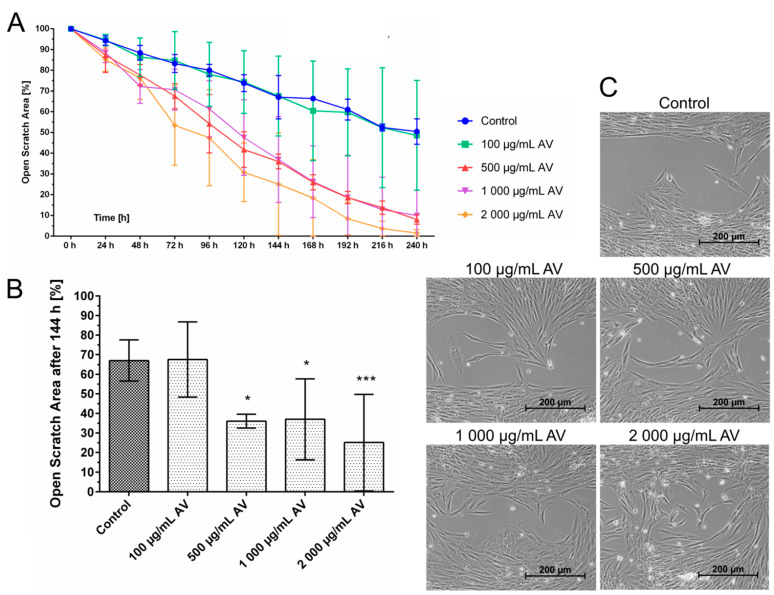
Effect of AV on cellular migration of fibroblasts. The scratch assay showed that concentrations of AV above 100 µg/mL significantly increased the migration of fibroblasts. (**A**) After 240 h the scratch area was still 50% open in the control group, while after treatment with 500 mg/mL, 1000 µg/mL, or 2000 µg/mL the AV gap was completely closed. (**B**) Open Scratch Area after 144 h. After 144 h, the scratch area of the control group was still open for 70% whereas high concentrations of AV above 500 µg/mL resulted in gap closure of 35%. Two-way ANOVA, comparison of each treatment group with untreated control group, N = 3 with fibroblasts from different patients, * *p* < 0.05, *** *p* < 0.001. (**C**) Representative images of the gap closure in the different treatment groups after 144 h.

Cellular migration is one of the critically important functions of wound healing. It was studied using a standardized culture-insert dish-system. The experiments revealed that AV alone accelerated gap closure of the originally cell-free area by fibroblasts (Figure 3) indicating a stimulation of fibroblast migration. Gap closure occurred faster with increasing AV concentration. After 240 h, the gap was already closed using AV concentrations of 2000 µg/mL and almost closed (<10% open cell-free area) using 500–1000 µg/mL, whereas the gap was still open around 50% after 240 h in the control group.

**Figure 4:**
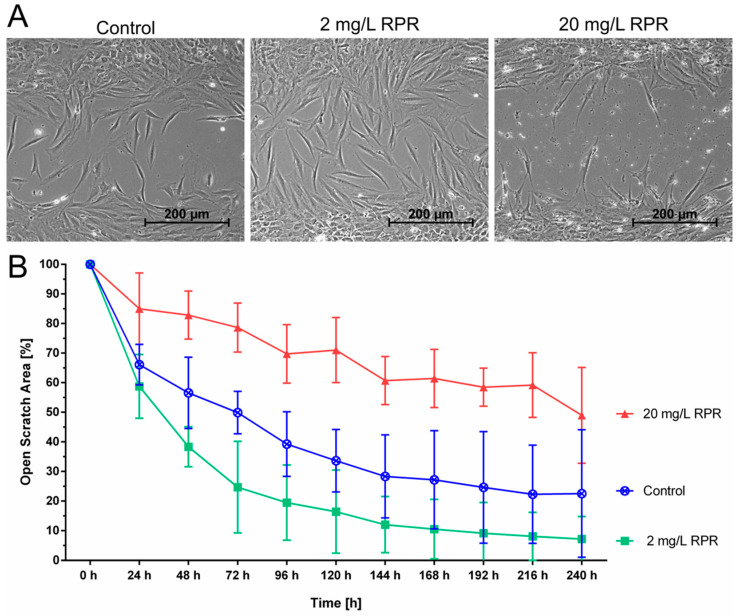
Effect of RPR on cellular migration of fibroblasts. The scratch assay showed that 2 mg/L of RPR increased the migration of fibroblasts, whereas 20 mg/L RPR decreased fibroblast migration. (**A**) Representative images of the open scratch area in the different treatment groups after 72 h. Treatment with 2 mg/L RPR resulted in nearly complete closure of the scratch area within 144–240 h, whereas gap closure did not occur even after 240 h with 20 mg/L RPR. (**B**) After 72 h, the scratch area of the control group remained 50% open. Low concentration of 2 mg/L of RPR reduced the open scratch area to 25%. With high concentrations of 20 mg/L RPR the scratch area remained >75% open after 72 h. N = 3 with fibroblasts from different patients.

The effect of different concentrations of RPR, namely 2 mg/L and 20 mg/L, on fibroblast migration was also tested. Higher concentrations of RPR of 20 mg/L slowed down gap closure, but interestingly, low concentrations of 2 mg/L accelerated gap closure compared to untreated control cells as shown in Figure 4.

**Figure 5:**
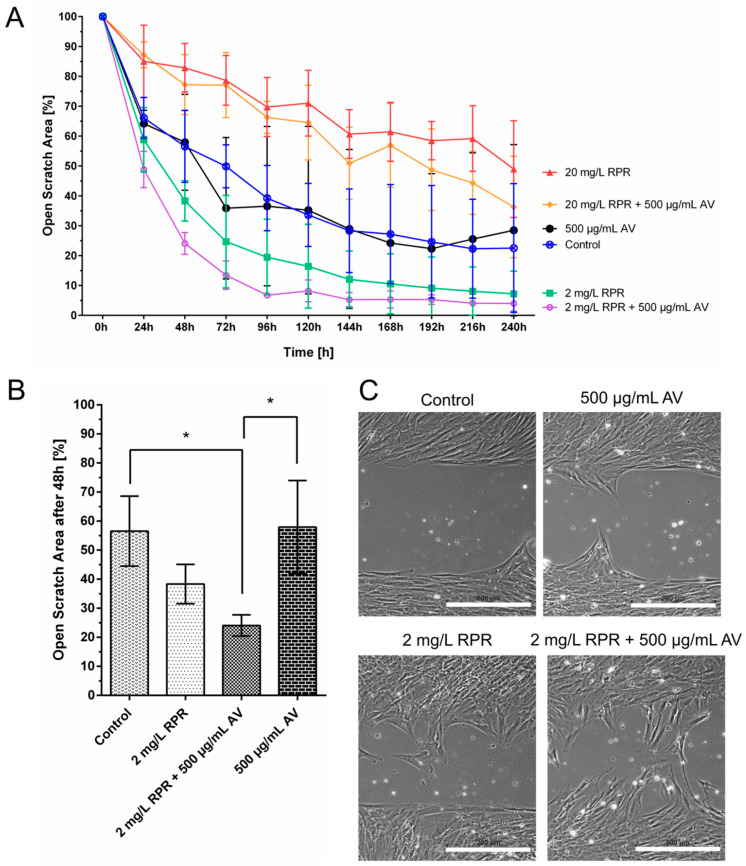
Effect of AV, RPR and their combination AVRPR on cellular migration of fibroblasts. The scratch assay showed that the combination of AVRPR significantly increased the migration of fibroblasts. (**A**) Treatment with 2 mg/L RPR or a combination of 2 mg/L RPR and 500 µg/mL AV resulted in nearly complete closure of the scratch area within 96–120 h. The combination of 2 mg/L RPR and 500 µg/mL AV resulted in fastest gap closure. In this treatment group the gap was already closed more than 90% after 96 h. In contrary, at that time point the scratch area in the control group was still 35% open. (**B**) Open Scratch Area after 48 h. After 48 h, the open scratch area of the control group remained >50% open. The combination of 2 mg/L RPR and 500 µg/mL reduced the open scratch area to <25%, whereas the gap remained >40% open if 500 µg/mL AV was used alone. Thus, the combination AVRPR significantly improved gap closure compared to AV alone and compared to the untreated control group. One-way ANOVA, comparison between all groups, N = 3 with fibroblasts from different patients, * *p* < 0.05. (**C**) Representative images of the open scratch of the control group and 2 mg/L RPR/500 µg/mL AV after 48 h. Scale = 200 µm.

Finally, AV and RPR were combined in the concentrations that were seen to be favourable for cellular migration when tested individually, namely 500 µg/mL AV and 2 mg/L RPR, the combination being denoted by AVRPR. As shown in Figure 5, gap closure occurred even faster if AV was used in combination with low concentrations of RPR compared to treatment with AV alone (48 h, *p* < 0.05). Gap closure (<10% open scratch area) was already nearly complete after 144 h when cells were treated with 2 mg/L RPR. The combination of 2 mg/L RPR and 500 µg/mL AV accelerated wound closure even further and gap closure already occurred after 96 h (Figure 5).

#### Calculation of the Synergistic Action of AV and RPR on Fibroblast Migration

The combination AVRPR showed synergistic effects on fibroblast migration in vitro. From the definition of pCI_AV_ and pCI_RPR_ given in 2.8. and SI; the curves in Figure 5A, as well as in both scenarios (Figure S4a,b), were strictly monotonic decreasing and did not intersect. Because 2 mg/L RPR + 500 µg/mL AV showed much lower values for all timepoints, both pCI-ratios, pCI_AV_ and pCI_RPR_, were strictly <1 by elementary analysis [36]. Calculation at for example t = 168 h yielded pCI_AV_ = 5.34/24.23 = 0.22 and pCI_RPR_ = 5.34/10.53 = 0.51. According to the experimental setup, quotients < 1 are sufficient to assume synergy.

### 3.4. Cell Cycle Analysis

**Figure 6:**
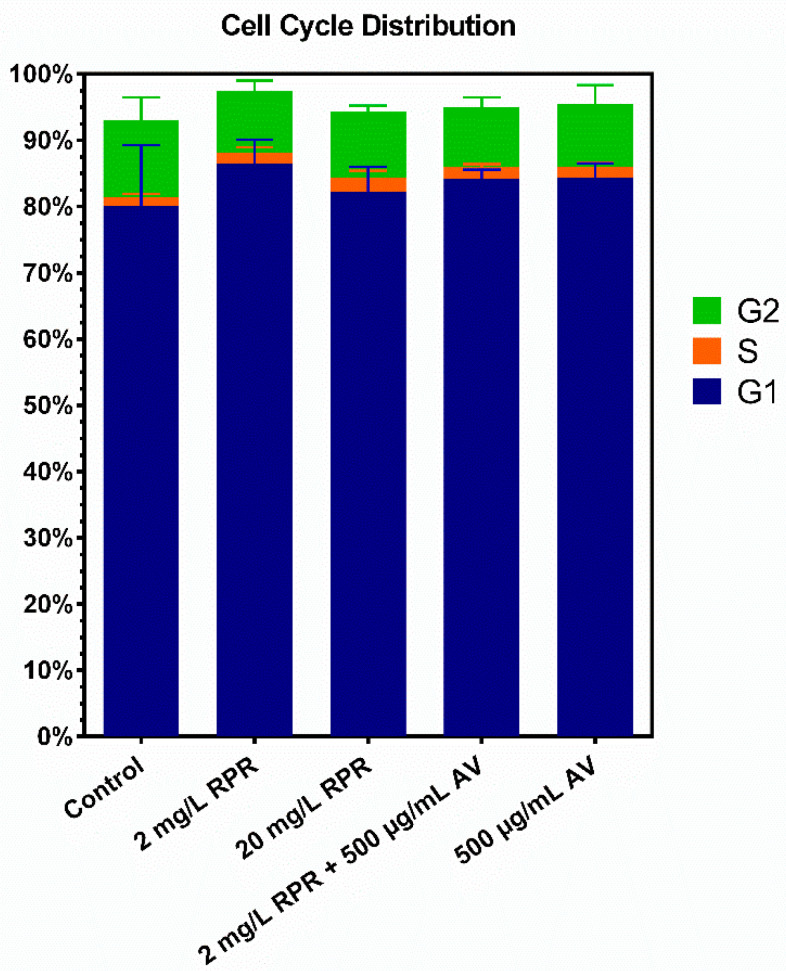
Cell cycle distributions of fibroblasts after treatment with AV and RPR. Neither RPR alone, AV alone, nor the combination of AVRPR significantly influenced the cell cycle distribution. N = 3.

To clarify the influence of RPR, AV, and their combination AVRPR on proliferation and cell cycle distribution, a cell cycle analysis was performed. As shown in Figure 6, neither RPR, AV, nor their combination influenced the cell cycle distribution significantly.

### 3.5. Actin Staining

Cell migration is associated with the remodelling of the cytoskeleton. During migration—among other effects—newly polymerized actin filaments push the cell membrane outwards forming lamellipodia and filopodia. To investigate whether AV and RPR exert effects on the actin network of fibroblasts we used fluorescent-labelled phalloidin, which binds to polymerized f-action in order to detect differences in the organisation of the actin cytoskeleton following treatment with AV and/or RPR. Actin staining was performed as an explorative single experiment to get a first notion of how the cytoskeleton and the morphology of fibroblasts might be influenced by AV and RPR and whether reorganization of the actin cytoskeleton could possibly contribute to the observed differences in the migratory capacity of the fibroblasts after treatment.

**Figure 7:**
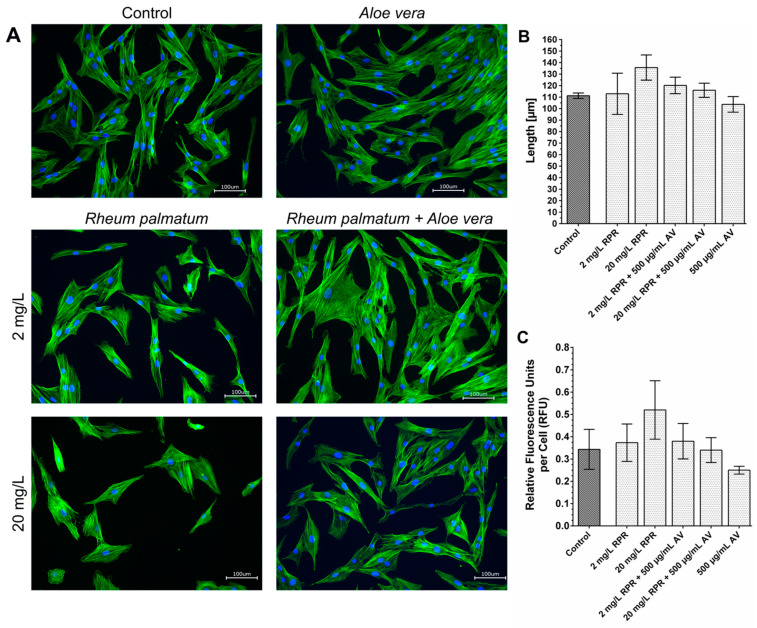
Actin staining of fibroblasts with phalloidin and evaluation using ImageJ. Staining of the actin with phalloidin revealed that the distribution of actin in fibroblasts treated with AV and/or RPR was not influenced compared to untreated control cells (**A**). One could only see a very slight trend that the fibroblasts treated with RPR appeared to be a little more elongated. Actin staining is shown in green, and nuclei are shown in blue. Scale = 100 µm, N = 1. Neither the average length of the fibroblasts (**B**), nor the intensity of the fluorescent actin staining per cell (**C**) were significantly influenced by treatment with RPR and/or AV. With one-way ANOVA, comparison of each treatment group with the untreated control group and fibroblasts from one patient, no significant differences were found.

Actin staining revealed no differences in the actin distribution within the fibroblasts in response to treatment with AV and/or RPR compared to untreated control cells. The average length of the fibroblasts was measured and the intensity of the actin staining per cell was quantified, however, no significant differences were found between the different treatment groups (Figure 7).

### 3.6. Broth Microdilution Assay

MIC was determined using the broth microdilution method. The combination of AVRPR showed a MIC of 2 mg/L against *P. gingivalis*, whereas AV alone showed no antimicrobial activity. RPR alone showed a MIC of 4 mg/L (Table 1).

#### Calculation of the Synergistic Antimicrobial Action of AVRPR

The combination AVRPR resulted in an FICI value of 0.48 against *P. gingivalis* which is defined as synergy (FICI ≤ 0.5) [42] as described in Section 2.12.

## 4. Discussion

This study was designed to identify plant derived substances suitable for oral topical use in patients with compromised mucosal wound healing. In brief, our study revealed that the combination AVRPR showed synergistic interactions on fibroblast migration and thus may have beneficial properties for wound healing. AV, RPR and their combination AVRPR enhance cellular migration while cell cycle distribution and thus fibroblast proliferation were not affected. Our study furthermore confirmed synergistic antibacterial activity against *P. gingivalis* for the combination AVRPR.

Interestingly, the same concentrations of AVRPR which exhibited antibacterial effects against *P. gingivalis* showed positive effects on fibroblast migration, which is relatively uncommon for antibiotics or antiseptics since cytotoxic effects are frequently seen in these agents [43,44]. Antiseptics are known for their broader antimicrobial spectrum, but on the other hand are known to be more cytotoxic than antibiotics [45]. Nevertheless, both substances may impair wound healing [46]. One of the major bioactive components of the RPR extract used herein are anthraquinones as confirmed by HPLC, these may contribute not only to the antimicrobial efficacy but at the same time promote fibroblast migration. Anthraquinones are derivates of anthracenes from the quinone group which are known to improve wound healing and possess antibacterial activity [34,47].

Analysis of the cellular viability revealed a broad spectrum of biocompatibility of AV ranging from 10–2000 µg/mL. Furthermore, high concentrations of AV from 500 µg/mL onwards were able to slightly stimulate the metabolic activity of fibroblasts. This is in line with previous studies, which showed stimulation of keratinocyte viability by AV [48].

In this study, we were able to show that AV and RPR were able to positively influence fibroblast migration. In contrast to untreated human fibroblast which did not fully close the scratch assay gap after 240 h, treatment with AV resulted in complete gap closure. AV in high concentrations of 2000 µg/mL was able to accelerate migration, which is in line with previous studies [48,49]. In our setup, cell culture medium without FCS was used to assess the effects on fibroblast migration without stimulating the cells to proliferate by FCS. In contrast to previous studies performed with fibroblast by Teplicki et al. and Shafaie et al., AV had no proliferative effect on fibroblasts in our study [48,49].

Cytotoxicity of RPR in high concentrations is well-described in the literature, for example in different human carcinoma cells [50,51], but not in human oral epithelial cells [35]. Here we found that RPR concentrations up to 100 µg/mL were largely biocompatible in fibroblasts with only minor effects on the cellular viability. Surprisingly, treatment with 2 mg/L RPR or a combination of 2 mg/L RPR and 500 µg/mL AV (AVRPR) resulted in nearly complete closure >90% of the scratch area within 96–120 h and AVRPR accelerated gap closure 2.5-fold compared to control. Thus, we are the first to be able to show that low RPR concentrations are biocompatible and have pro-migratory properties.

Neither RPR or AV alone, nor the combination AVRPR significantly influenced cell cycle distribution in our study. Hong et al. reported induction of G0/G1 cell cycle arrest and apoptosis for an isolated anthraquinone of RPR on human breast cancer cells and thus showing antiproliferative effects [52]. However, human primary fibroblasts may not be directly comparable to cancer cell lines in terms of their reaction towards different substances and as mentioned above, higher concentrations were used in those experiments. The important information from this experiment in a wound healing setting is that the combination of the two investigated substances is probably not able to induce excessive fibroblast proliferation in the scope of fibrosis [53,54].

Staining of the actin-fibers with phalloidin revealed no differences in the actin distribution in response to treatment with AV and RPR. However, the staining was only performed once, and it was performed with stationary cells. Possibly, staining of cells during migration and closure of the originally cell-free area in the scratch assay would have revealed differences in the actin network in response to treatment with AV and RPR. Prospectively, it would also be interesting to investigate other cytoskeletal proteins besides actin in further experiments.

In this study, AV alone did not show antibacterial activity against *P. gingivalis* as judged by broth microdilution assay. The antibacterial effect of AV was previously reported to be dose-dependent, but no absolute concentration was given in a study performed by Jain et al. since AV gel was prepared from fresh AV without analyzing the components [55]. Another study found MIC values for AV of 50 µg/mL on *P. gingivalis* strain ATCC 33,277 [37], which could not be replicated in our study. Both above-mentioned studies used fresh AV plants for antimicrobial tests. The found inhibitory activity may derive from low concentrations of Aloe-emodin, which may be present in *Aloe vera* leaves [56]. Due to the antimicrobial effects of anthraquinones, the antibacterial effect of AV in the above-mentioned study may have been derived from Aloe-emodin which may have been present in higher quantities in freshly prepared AV. However, in our study spray-dried AV powder without relevant concentrations of Aloe-emodin was used, which may have resulted in this discrepancy.

Antibacterial properties of RPR have been previously reported [34,35,36,57] and suppression of planktonic growth of *P. gingivalis* was reported to start at 3.9 mg/L. Our study revealed a MIC of 2 mg/L against *P. gingivalis* for the combination AVRPR [36]. MIC for AV alone was >2048 mg/L and 4 mg/L for RPR was found in a previous study by the author [34]. Therefore, the combination of AVRPR with a MIC of 2 mg/L showed synergistic effects as calculated with an FICI of 0.48. Synergistic effects as calculated by pCI were sufficient to assume synergies of AVRPR and show the effects of faster closure in comparison of the extracts. These effects even occurred if migration inhibiting concentrations of RPR (20 mg/L) were combined with AV (500 µg/mL).

Fibroblasts from different patients reacted differently to the same treatment, especially regarding AV concentrations ≥1000 µg/mL (Figure 1) and 500 µg/mL (Figure 3A and Figure 5A). Furthermore, the influence of AV, RPR, and AVRPR on fibroblast migration was different in the samples (Figure 5 and Figure S4a,b). Due to the experimental setup of this laboratory study, we could neither analyze the cause–effect relationships nor do more in-depth modeling of the causality of the effects and mechanisms of action, but this will be subject to further experiments.

The authors of this study hypothesize that the synergistic effect of AVRPR could be attributed to acemannan, the main bioactive polysaccharide in the AV extract used herein. Acemannan, a β-(1,4)-acetylated water-soluble polymannose is considered a biologic response modifier [58,59]. The long sugar chains in acemannan may aid cells to adhere during migration. Acemannan showed immunomodulatory effects on gingival fibroblasts in vitro [60]. In male Sprague Dawley rats, acemannan increased fibroblast proliferation and stimulated KGF-1, VEGF and type I collagen expression [61]. In another in vivo study, acemannan increased proliferation of bone marrow stromal cells, VEGF, BMP-2, alkaline phosphatase activity, bone sialoprotein and osteopontin expression, and mineralization in male Sprague Dawley rats [62]. These findings suggest, that acemannan plays a role in oral soft and hard tissue wound healing, but further studies are needed to clarify the exact molecular mechanisms.

The observed results may contribute to improving current therapeutics for the topical treatment of wound healing disorders. In particular, this may be interesting for patients suffering from radiation induced mucositis, since RPR is used in an ointment for burn wounds according to the Chinese Pharmacopoeia (Committee of the Pharmacopoeia of PR China, 2015) and has shown beneficial results for this indication in animal models [63].

## 5. Conclusions

Our study revealed that RPR and AV synergistically in combination (AVRPR) possess antibacterial properties and accelerate fibroblast migration in vitro. These findings, suggest the combination of both plant extracts for the improvement of oral tissue regeneration in patients with a compromised wound healing capacity.

## 6. Patents

A European patent (EP22190349.5) was filed on 15 August 2022.

## Figures and Tables

**Table 1:** Effect of the combination AVRPR on *P. gingivalis* growth. The broth microdilution assay revealed synergistic antibacterial effects if RPR and AV are combined to AVRPR. AV alone showed a MIC of >1024. RPR alone showed a MIC of 4 mg/L which would be expected for the combination AVRPR if an additive effect existed. The MIC of AVRPR was 2 mg/L indicating a synergistic effect. N = 3.

Substance	MIC
AV	>2048
RPR	4
AVRPR	2

## Data Availability

Not applicable.

## Supplementary Materials

The following supporting information can be downloaded at: https://www.mdpi.com/article/10.3390/pharmaceutics14102060/s1, Figure S1: HPLC chromatogram of the aloe vera sample; Figure S2: Full (upper) and zoomed-in (lower) 1H-NMR spectra of aloe extract (600 MHz, D2O); Figure S3: Full (upper) and zoomed-in (lower) 13C-NMR spectra of aloe extract (151 MHz, D2O); Figure S4: (a) Effect of the combination of RPR and AV on cellular migration of fibroblasts. Displayed are FB1819 and FB2419 without FB0719. The scratch assay showed that the combination of RPR and AV significantly increased the migration of human primary fibroblasts. (b) Effect of the combination of RPR and AV on cellular migration of fibroblasts; Table S1: Aloe vera content as confirmed by HPLC analysis (provided by vendor).
