# Supplementary material for: Extracts of Rheum palmatum and Aloe vera Show Beneficial Properties for the Synergistic Improvement of Oral Wound Healing
Source: Pharmaceutics. 2022 Sep 27;14(10):2060. doi: 10.3390/pharmaceutics14102060 (PMC9610717; doi:10.3390/pharmaceutics14102060)
Supplement: Supplementary file 1 [file pharmaceutics-14-02060-s001.zip › Figures S1-S4.pdf]

## HPLC (high-performance liquid chromatography) analysis

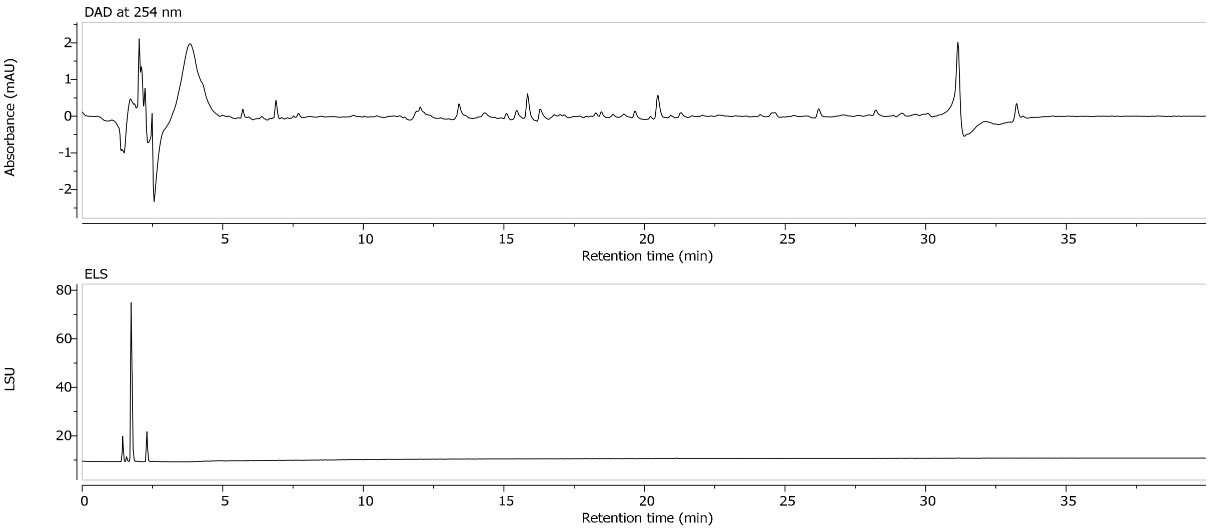


Figure S1. HPLC chromatogram of the aloe vera sample. Shown are the traces of a diode array detector (DAD) monitored at 254 nm and an electrophoretic light scattering (ELS) detector.

## NMR (nuclear magnetic resonance)

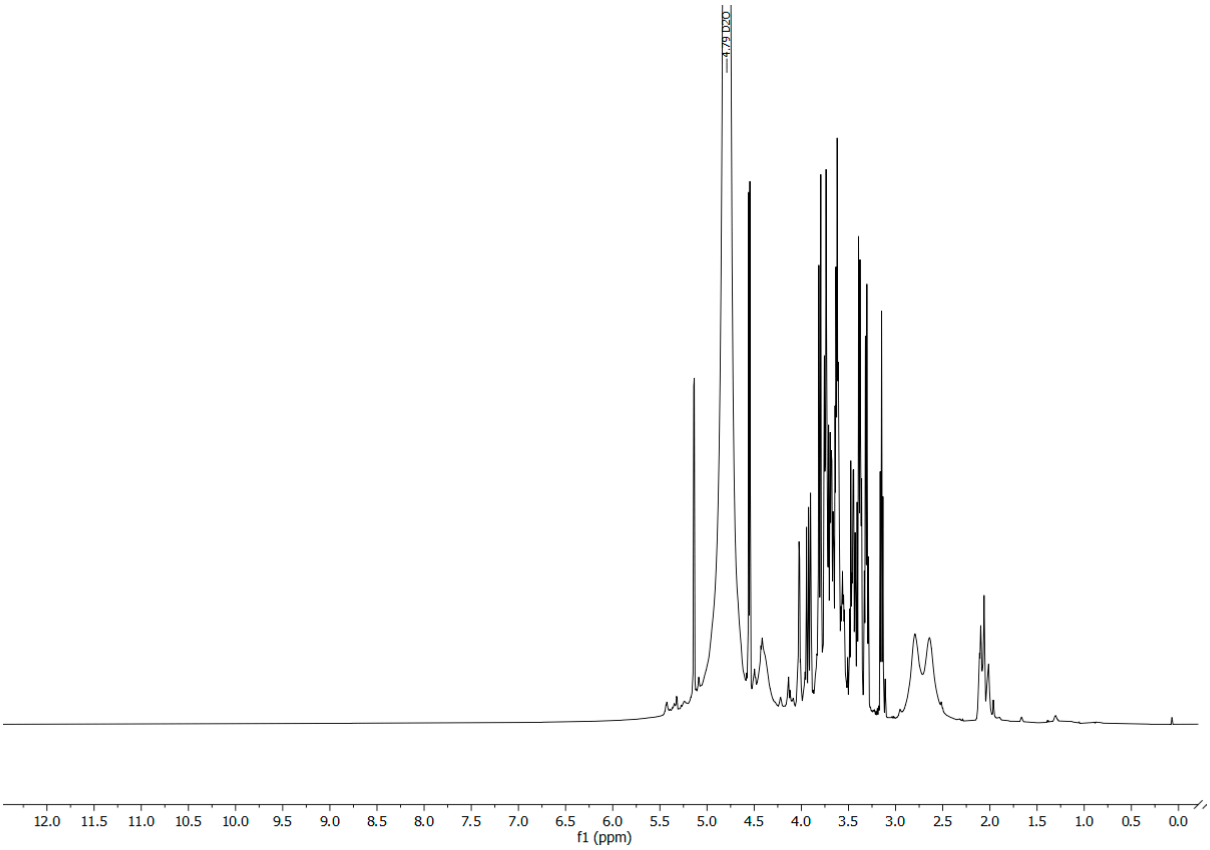

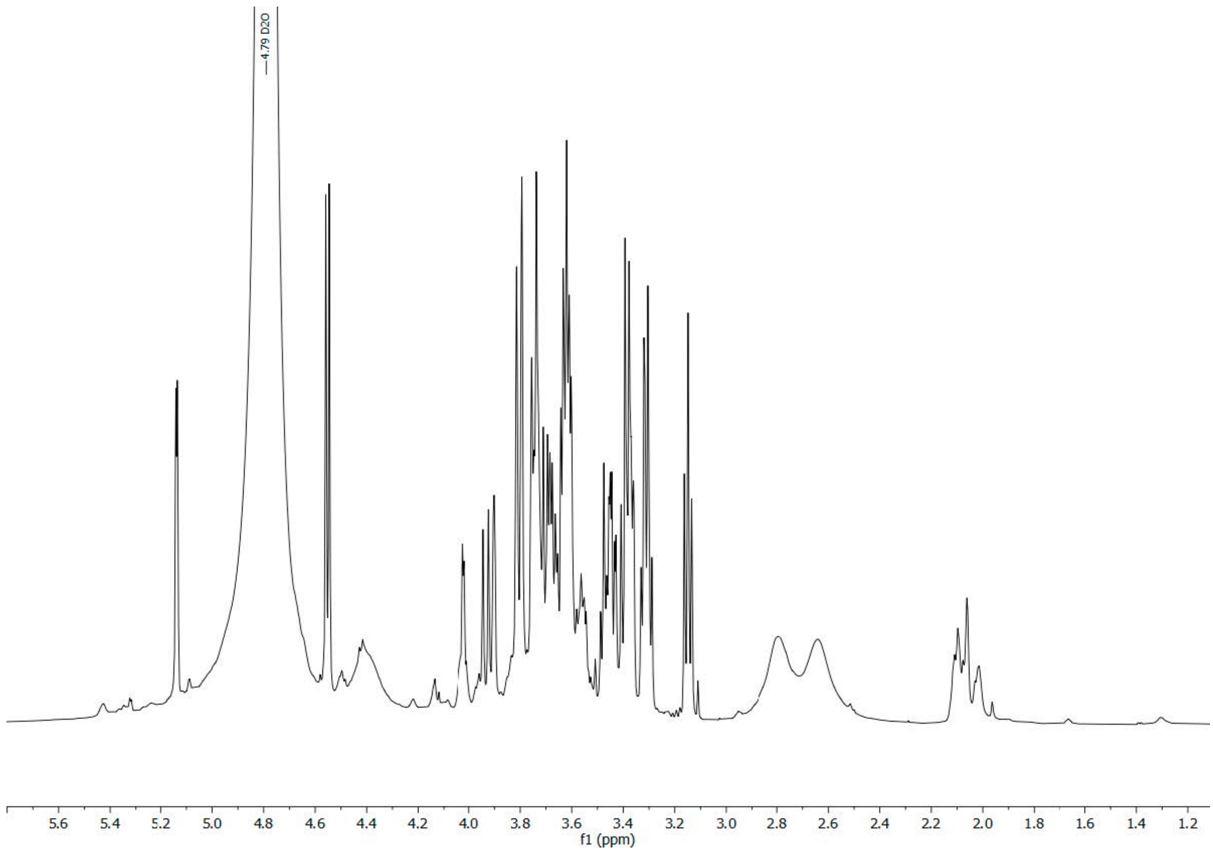


Figure S2. Full (upper) and zoomed-in (lower) $^{1}H$-NMR spectra of aloe extract (600 MHz, $D_2O$).

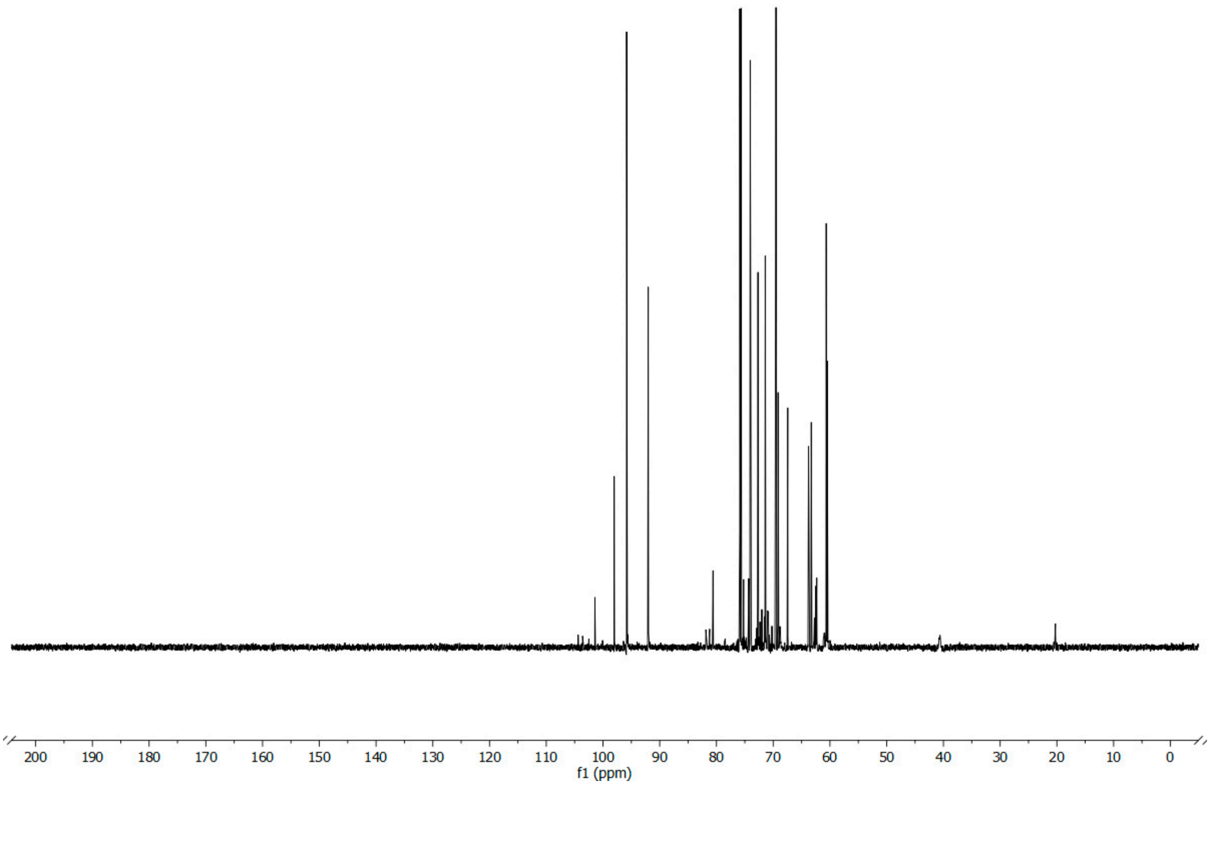

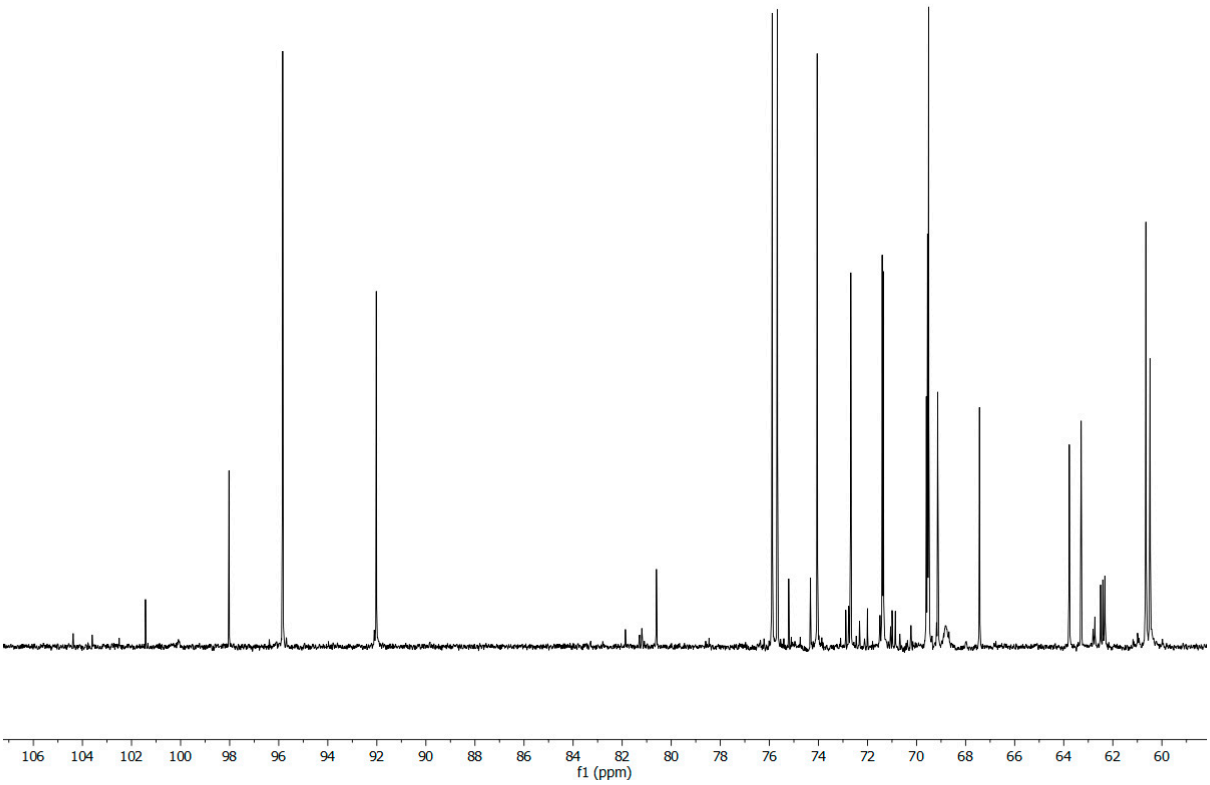


Figure S3. Full (upper) and zoomed-in (lower) $^{13}C$-NMR spectra of aloe extract (151 MHz, $D_2O$).

## pCI

To establish and determine notions of synergy and antagonism [35], a method based on median effect principles was established and derived from generalized mass action considerations. By fundamental considerations of dose-response results of physicochemical and biochemical systems, a uniform method has been established, which allows an analysis in simple as well as in complex biological systems.

Due to the experimental approach chosen here and with focus on the observable effect gap closure of the standardized cell culture as a temporally extended series of measurements, first a phenomenological approach was chosen, which is based on Chou et al. [35], but thereby skipping the "median effect plot". On the one hand, the requirement $f_a + f_u = 1$, i.e. the disjoint decomposition of 1 = 100% could not be implemented directly in our setup. If necessary, the observables would have to be normalized phenomenologically to 1. On the other hand, the conditions 1. and 2.a and 2.a - 2.c mentioned in [35] p. 31/32 section "Requirements for Analyzing Multiple Drug Effects" can be fulfilled only to a limited extent because of the experimental complexity. This is especially due to the clinical setup and the preparation of the test scenarios:

- With regard to toxicity, only appropriate and not all doses were approved for time series analysis of cell cultures.
- The ingredients applied to the samples are not single active ingredients applied directly to the (punctiform/local) site of action, but in both cases "AV" and "RPR", thus also in the "AVRPR" combination, are without exception plant extracts and therefore combinations of active ingredients. The extraction or analysis of individual effects is a priori not experimentally accessible, only phenomenological effects are observable.
- Furthermore, the plant extracts "AV", "RPR" and "AVRPR" are prepared and applied homogeneously to the samples over a wide area. They therefore require the additional consideration of their preparation and the broad physical application of the active ingredient to the sample(s). As a supportive phenomenological simplification we assume the same properties in all three cases of application as well as in the fourth case of non-application, so that we may suppose that these effects are identically distributed in a quotient formation of the observed effects/measurements and numerically divide out (i.e., any necessary modifications of the three plant extracts are identical in all four cases, each of the possible quotients from this comparison of sample geometry is 1).
- The samples on which the active substances were applied to standardized sections have the same cell behavior within the sample geometry/arrangement and application of the cut, and they have the same behavior over the entire observation period, i.e. at each measurement time point, it is assumed that the samples behave in the same way and that these properties of the samples are also described by parameters which divide out by quotients of the measured values.
- These aspects of extracts as well as of the samples cannot be described by an analytical treatment of individual effects or temporal/causal modeling, but can be understood only by the fundamental conception a priori as "systems of extracts" (i.e. systems of combinations of single agents) and their application to "systems of samples". Therefore currently only the assessment of observable effects, but not of strict causal mechanisms or methods can be made.

Consequently, according to Chou et al. we were guided by the "Equations for the Effects of Multiple Drugs" and define the measurement of gap closure as an observable effect. The measurement ("gap closure") is made in absolute values ("length measurement") relative to the sample geometry, i.e. of the spread cells and the standardized identical sections applied to them. The given values of the four measurement series ("untreated/control", "AV", "RPR" and "AVRPR") correspond to the percentage of gap closure, intrinsically normalized to the uniformly equal basic section(s) in the cell arrangement. A quotient formation between the effect with application of an active substance ("$f_a$") compared to the effect without application of an active substance ("$f_u$") thus relates to a mesurement of the impact, since according to our above assumptions the phenomenological descriptions of the combination and the sample geometry divide out against each other. This impact is thus to be interpreted directly interpreted as the quotient "$f_a/f_u$", which bridges parts the gap to reference [35]. Thus, the effects/impacts are phenomenologically described by the quotients "$f_{AV}/f_u$", "$f_{RPR}/f_u$" and "$f_{AVRPR}/f_u$" in percentages, where "$f_u$" represents the value of the untreated sample. Accordingly these quotients can also be directly related to each other, whereby the untreated values "$f_u$" divide out in each case of quotient formation and thus do not appear explicitly in the results or the ratios. This simplifies also the phenomenological comparison of the values at each (common) measurement point of the time series and enables direct comparisons of such ratios by defining a "phenomenological Combination Index" according to

$$pCI_{AV} := f_{AVRPR}/f_u \ / \ f_{AV}/f_u = f_{AVRPR}/f_u * f_u/f_{AV} = f_{AVRPR}/f_{AV}$$

resp.

$$pCI_{RPR} = f_{AVRPR}/f_u \ / \ f_{RPR}/f_u = f_{AVRPR}/f_u * f_u/f_{RPR} = f_{AVRPR}/f_{RPR}.$$

Values of pCI smaller than 1 thus show the effects of faster closure in comparison of the extracts and thus a better phenomenological efficiency under the above assumptions.

Accordingly, the observed effect of the combined extracts ("AVRPR ") of the measurement series is in each case better than the observed individual effects for the application of AV only and RPR only, even if the strict definition of synergy in the sense of Chou et al. due to the phenomenological determination of the effects (by measurement of the gap width) is not directly applicable (see above). If one wants to consider *both* time courses of the effects of the individual extracts and put them into relation to the combination, one can use as further phenomenological indices:

$$pCI_2 = f_{AVRPR}/\min(f_{AV}, f_{RPR})$$

which, relative to $pCI_{AV}$ and $pCI_{RPR}$ does not introduce any really new insights, but essentially only a more compact notation by respecting the common minimum $\min(f_{AV}, f_{RPR})$ of both individual extract series. A step towards the original "Combination Index (CI)" given by Chou et al. can be established by the phenomenological definition

$$pCI_3 = f_{AVRPR}/f_{AV} * f_{AVRPR}/f_{RPR} = f^2_{AVRPR} \ / \ (f_{AV} * f_{RPR}),$$

which in contrast to the "linear" approaches with $pCI_{AV}$, $pCI_{RPR}$ and $pCI_2$ takes certain different function courses over the time into account and compensates different functional behaviour partially.
For all phenomenological approaches ($pCI_{AV}$, $pCI_{RPR}$ and $pCI_2$) it is possible to fit and represent the data curves by appropriate polynomials, so with sufficiently detailed statistics one can easily represent the above quotients analytically by polynomial division. Using exponential fits to the curve data instead of polynomials, the quotient may be expressed as well analytically, moreover this approach offers by logarithm extraction a direct linear comparison of the respective curve parameters. This last approach

– based on more experimental data – closes the gap phenomenologically to the logarithmic extractions of the median-effect plot and the median effect principle in [35].

It is important to emphasize that our approach does not make any statements about individual components or individual effects, but that applying "the AV system", "the RPR system" and "the AVRPR system" to a respective sample system prepared as identically as possible and compared to the same untreated sample system, the effects of the different applications - in each case normalized to the untreated system - are compared with each other and thus provide a phenomenological approximation to the more basic median-based Chou et al. method for discussion. Smaller values signal by definition better wound closure by AVRPR.

Two statistical scenarios are shown in Figure S4a and S4b. In scenario A (Figure S4a) calculation at t=168h yields $pCI_{AV}$=6.82/35.86=0.19 and $pCI_{RPR}$=6.82/14.89=0.46. The case scenario B (Figure S4b, single value) has to be treated separately and carefully due to the very small values, $pCI_{AV}$ = 2.38/0.98=2,43 and $pCI_{RPR}$ = 2.38/1.80 =1.32. If we estimate an error of ~ 1.5 in AV and RPR, synergistic or antagonistic effects cannot be decided statistically in the single case of fibroblast line FB0719 (Figure S4b, single value).

Due to the experimental design of the study, primary human fibroblast obtained from human gingiva of three different patients were used and they were pseudonymized under the names FB1819, FB0719 and FB2419. Equal treatment of these three samples (hereafter Evaluation A) lead to a statistical sample which, when calculating the mean value and the standard deviation according to classical patterns to very large standard deviations, in many cases in the amount of 50-80% of the mean value. To improve this statistically unsatisfactory situation and to reflect laboratory observations during the experiment, we have postulated and formulated the conjecture given below, which is due to the conspicuously improved curability of the sample FB0719 relative to the other two samples FB1819 and FB2419. In order to further support this hypothesis, the samples were grouped again within the random sample (FB1819 and FB2419 vs. FB0719), and for the two first-mentioned cell cultures the mean and standard deviation were determined separately (Figure S4a and S4b). Due to the experimental setup and few data, care has to be taken when dividing the small values for large observation time.

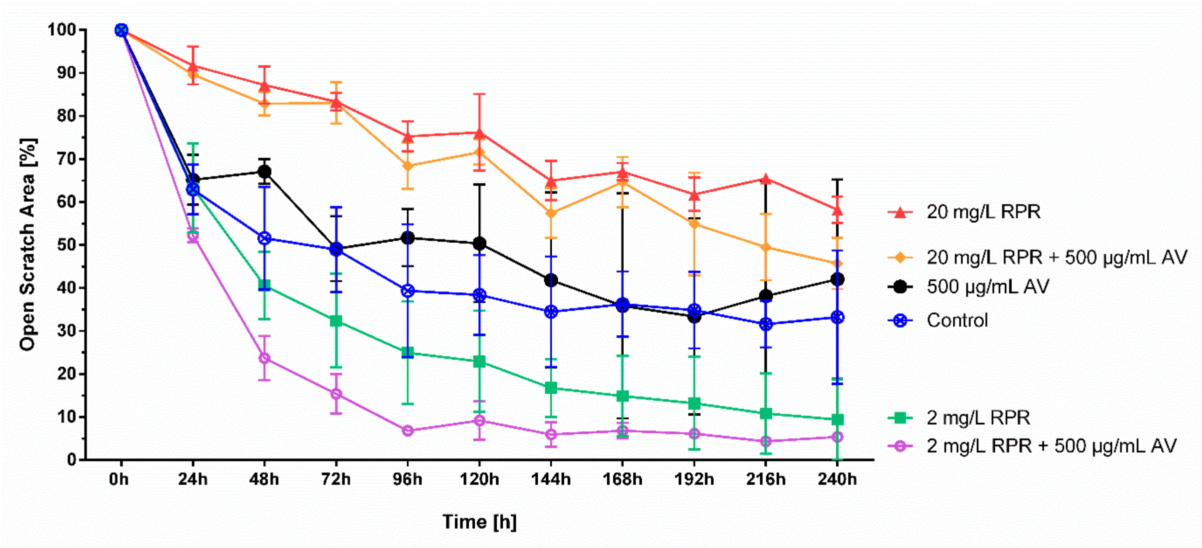


(a)

.

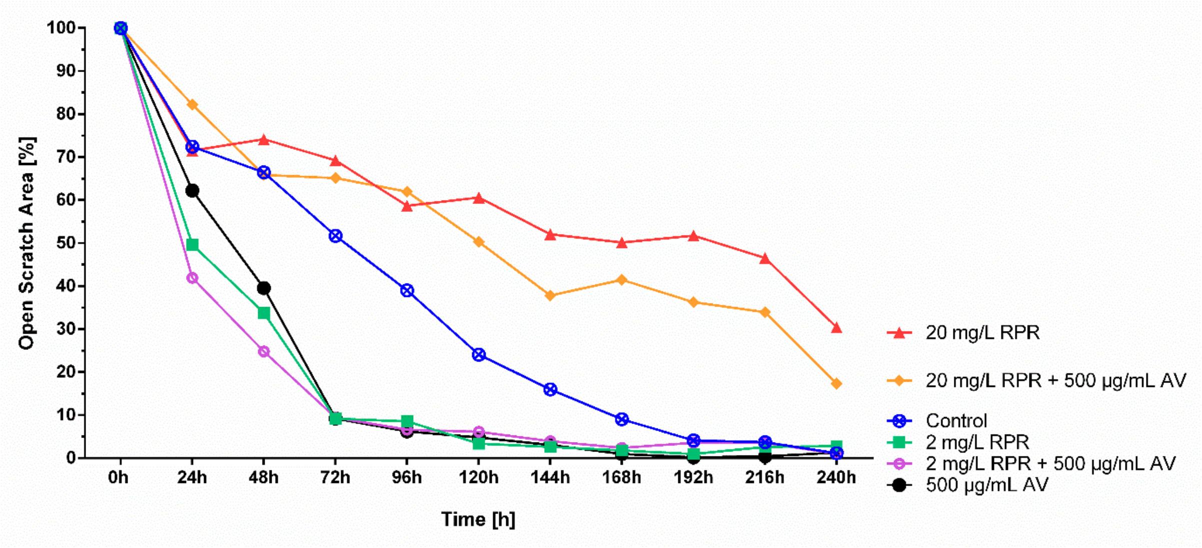


(b)

Figure S4. (a) Effect of the combination of RPR and AV on cellular migration of fibroblasts. Displayed are FB1819 and FB2419 without FB0719. The scratch assay showed that the combination of RPR and AV significantly increased the migration of human primary fibroblasts. (b) Effect of the combination of RPR and AV on cellular migration of fibroblasts. Displayed is FB0719 without FB1819 and FB2419. The scratch assay showed that the combination of RPR and AV increased the migration of human primary fibroblasts, but closure occurred faster with AV and AVRPR compared to FB1819 and FB2419.

Literature:


35. Chou, T.C.; Talalay, P. Quantitative analysis of dose-effect relationships: the combined effects of multiple drugs or enzyme inhibitors. *Adv Enzyme Regul* **1984**, *22*, 27-55, doi:10.1016/0065-2571(84)90007-4.
