# Supplementary material for: Extracts of Rheum palmatum and Aloe vera Show Beneficial Properties for the Synergistic Improvement of Oral Wound Healing
Source: Pharmaceutics. 2022 Sep 27;14(10):2060. doi: 10.3390/pharmaceutics14102060 (PMC9610717; doi:10.3390/pharmaceutics14102060)
Supplement: Supplementary file 1 [file pharmaceutics-14-02060-s001.zip › Table S1.pdf]

Table S1: Aloe vera content as confirmed by HPLC analysis (provided by vendor).

	Content (%)	Content (mg/L)	Origin of component
Aloverose (polysaccharide)	14.0	977.1	fresh Aloe Vera
Glucose	22.9	1605.4	fresh Aloe Vera
Malic acid	20.9	1463.5	fresh Aloe Vera
Lactic acid	0.1	7.7	degradation (bacterial)
Citric acid	1.0	71.1	WLM or added acidifier
WLM	Traces		whole leaf marker (WLM)
Fructose	Detected		fresh Aloe Vera
Mg	0.7	46.9	fresh Aloe Vera
Ca	2.0	137.8	fresh Aloe Vera
Maltodextrin	Not detected		formulation aid for drying
Acetic acid	Detected		degradation (hydrolysis)
